# Novel Method of Electromagnetic Field Measurements of the Human Brain

**DOI:** 10.7759/cureus.21982

**Published:** 2022-02-07

**Authors:** James Wiginton, James Brazdzionis, Tye Patchana, James Hung, Yongming Zhang, Dan E Miulli

**Affiliations:** 1 Neurosurgery, Riverside University Health System Medical Center, Moreno Valley, USA; 2 Electrical Engineering, Quasar Federal Systems, San Diego, USA; 3 Medical Physics, Quasar Federal Systems, San Diego, USA; 4 Neurosurgery, Arrowhead Regional Medical Center, Colton, USA

**Keywords:** magnetic field sensing, magnetic field, neuroimaging, medical imaging, neurosurgery, electromagnetic field

## Abstract

Introduction

Advancements in neuroimaging have changed the field of medicine. Computed tomography (CT) and magnetic resonance imaging (MRI) typically produce a static image of the brain, while continuous electroencephalogram (EEG) data is limited to the cortical surface. The brain’s chemical reactions produce an electric circuit that generates a magnetic field. We seek to test the ability of a non-contact sensor to measure the human brain’s electromagnetic field (EMF).

Methods

A lightweight, inexpensive construct was designed to hold EMF sensors to non-invasively measure the human brain’s dynamic EMF. Measurements were conducted on non-clinical human volunteers. Background data without the human subjects was obtained, followed by introducing human subjects. Motionless human subject data was obtained, followed by a subject performing a task. Finally, a subject received auditory stimulation, and data was obtained.

Results

Our non-contact sensor was able to detect a difference between background activity without a human subject and the electromagnetic field of a human brain within the scalp and skull. Detectable differences in magnetic field potential were also obtained when the subject performed a task and received auditory stimulation.

Conclusion

It is possible to continuously measure living human brain dynamic electromagnetic fields throughout the entire brain in a non-contact, non-invasive, continuous manner through the human scalp and skull in the standard environment. The signals are unique to the individual human and can be differentiated from background activity.

## Introduction

The advancements in neuroimaging over the past few decades have changed the field of medicine, neurology, and neurosurgery. The ability to non-invasively peer inside the fortified skull with structural and functional imaging techniques such as computed tomography (CT), magnetic resonance imaging (MRI), and electroencephalogram (EEG) has expanded our understanding of the human brain exponentially. Structural imaging obtained by CT and standard MRI has allowed neurosurgeons to localize pathology and make surgeries safer for patients. However, these modalities only provide a static image of the brain, and the functional data provided by EEG is limited to the cortical surface [1]. Functional MRI (fMRI) is a method whereby functional data can be obtained; however, the measurement method detects changes in blood flow. This is an attempt to associate the function with associated physiologic changes rather than directly measuring neural signaling [2].

The nervous system is an electrical circuit formed by chemical reactions with resultant electrical signals flowing to and from neurons. Any flowing electrical current generates an electromagnetic field (EMF) [3]. The source of the electrical currents is chemically communicating dendrites of the horizontally oriented pyramidal cells, which cause excitatory postsynaptic potentials, thereby generating a neural current flowing perpendicular to the cortical surface [1]. Recently it was shown by Carson et al. that neuronal tissue EMF from donor mice hippocampal tissue can be measured passively up to 3 cm away [4]. Given this knowledge combined with information from superconducting magnetoencephalography, our experiments sought to passively, continuously, and in real-time measure EMF emitted by the human brain at room temperature with a small, portable, user-friendly device. If the intrinsic magnetic field of the human brain can be measured passively, continuously, and directly, this may lead the way to a new form of neuroimaging technology based upon function. Furthermore, if it is possible to measure and understand the intrinsic activity of the human brain under normal physiologic conditions, it may be possible to record and subsequently alter pathologic magnetic fields in the diseased human brain. The goal of this study was to determine whether it is possible to directly measure the EMF in a human subject in a non-invasive, non-contact, and continuous manner.

## Materials and methods

Sensors and shielding

IRB approval was obtained from Arrowhead Regional Medical Center entitled “In-vivo non-contact remote measurement of neuronal activity” protocol #21-05. Proprietary passive electromagnetic field sensors (BS-1000) were designed and built by Quasar Federal Systems (San Diego, CA) and were used for all measurements. The sensors were 18 inches long and ¾ inch in diameter connected to amplifiers. The sensors were secured using zip-ties to polyvinyl chloride (PVC) cube, and the subjects were positioned within the cube surrounding the head. Various configurations were developed to determine the optimal positioning of sensors around the human non-clinical volunteer subject’s head, allowing for a wide range of orientations. The sensors did not touch the subject’s head and were up to 4.5 cm away from the scalp.

The subjects were placed outside and inside an electrically isolated room acting as a Faraday shield (electromagnetic shielding) with and without an additional enclosure of Mu-metal (MuMETAL®, Magnetic Shield Corporation, Bensenville, IL). The Mu-metal magnetic shield composition consists of nickel, molybdenum, silicon, manganese, and iron [5]. The sensors sat outside the Mu-metal enclosure of the cube and helmet. The sensors were at a gain setting F, with a 10x gain/2 kilohertz (kHz) gain/filter module. The ultra-low noise magnetic induction room temperature sensors have a detection sensitivity of 1 pT/rtHz at 1 Hz and capture magnetic response between 1 Hz and 2 kHz within the cylinder of detection.

Results were obtained by having the subjects lie flat on a rug and concrete slab with the head placed inside the PVC cuboid construct. A surrounding barrier of Mu-metal was used to decrease magnetic interference from the outside environment and the subject’s other sources of electromagnetic energy.

Data capture and analysis

All signals were captured at 5-kilo samples per second (ks/s) on a laptop computer using a 16-bit National Instruments Data Acquisition Card and LabVIEW software (National Instruments Corporation, Austin, TX). Data were post-processed with Igor® Pro 8 software (Wavemetrics Inc., Lake Oswego, OR). Frequency-domain data, also called Fast Fourier transform (FFT), of 120-second time-domain was processed, and the cumulative composite of the voltage recorded in 20-second bins versus frequency recorded over 120-second blocks was plotted. The data collection is binned into 0.3 Hz increments from 1 Hz to 2 kHz over 20 seconds. Therefore, at 5 ks/s, there were 100,000 data points per Hertz collected over 20 seconds. FFT processing allowed the accumulation of voltage amplitude over time to be collected at the desired frequencies measured. The frequency-domain graphs demonstrate detected magnetic fields in the chosen recorded frequency during the episode. We focused on responses from 0-20 Hz, but we collected signals up to 2,000 Hz. Therefore, 2 million data points were collected in 20 seconds. FFT performs a complex transformation of data and represents a summative average of data with increased frequency. This increased frequency identifies values that are reproducibly repeated more within the data set compared to other measured signals. Performing dedicated statistical analysis of FFT data is currently undergoing research in the statistical literature; therefore, analysis is only possible in a strictly observational fashion [6]. A graph of the sensor’s FFT curves was plotted for comparison. Waveform analysis occurred by independent investigators visualizing plots for comparison.

Tests

We sought to determine if dynamic human brain EMF signals could be reliably obtained from the passive, non-contact EMF sensors. Tests were performed with and without volunteer human subjects (see Table 1). The sensors were placed around the cube in different configurations and polarities as close to the subject’s head as possible without touching the hair or scalp to avoid interference. Tests were first performed without a human subject to obtain background results for a baseline, with the subject outside of the electrically insulated room (Faraday shield). This was accomplished with Test 1. For all tests, a right-sided, left-sided, and background sensor were used. Each sensor has a positive and negative polarity, and this was not kept consistent during initial tests. During the initial phases, the sensor would be placed with the positive end toward the subject and sometimes would be pointed away. However, after some initial inconsistency, the sensor always remained positive side toward the subject.

**Table 1:** Tests with and without human to determine sensor interference

Test	Description
1	Background without human subject
2	Background with human subject
3	Tapping on right leg with piezo on right leg
4	Background with human subject
5	Tapping on right leg with piezo on right leg exactly 50 times with period of no movement following
6	Background with human subject
7	Generate 0.5Hz, 10Vpp, 5ms pulse width audio pulse using a piezo film
8	Background without human subject

After background data was obtained in Test 1 without the human subject in place, the sensors remained in the same configuration for Test 2. The subject was introduced into the electrically shielded room, into the cube stabilizing the sensors, and remained motionless. Test 2 was to determine whether there is a different signal frequency and amplitude with a human subject by measuring the change from baseline without the subject.

Test 3 introduced a piezoelectric device that was secured to the subject’s right leg. The subject was asked to tap the device with their right index finger every 2 seconds, which allowed visualization of the exact interval of movement and its relation to Hz peaks in the results. In Test 4, Test 2 methodology was repeated to gain background data after Test 3 completion. In Test 5, tapping was repeated on the device 50 times, and then tapping was stopped to record “no tapping” data at the end of the same test. Test 6 represents another background data test with no subject movement.

In Test 7, a different non-magnetic piezo film was used to create a sound at the frequency of 0.5 Hz and 10 peak-to-peak voltage (Vpp) with 5-millisecond pulse width. Sensors captured data from the human subject while the piezo audio device emitted sound. Of note, the sound did not generate magnetic signals. Test 8 was then performed to obtain repeat background testing without the human subject.

## Results

**Figure 1:**
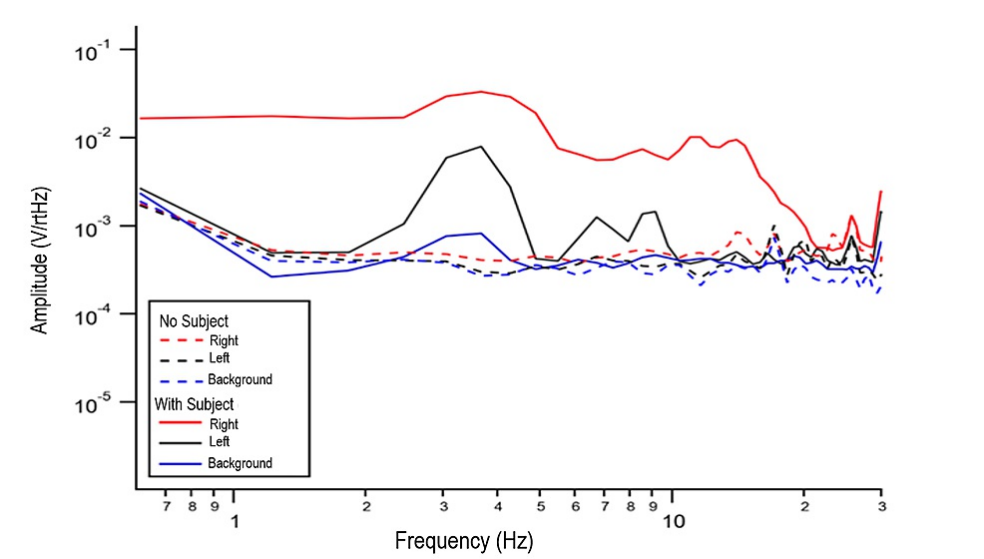
FFT comparison of Test 1 (no human subject) and Test 2 (with human subject) data. Dotted lines represent Test 1 data and solid lines represent Test 2 data. Right sided brain recording using red lines and left sided brain data using blue lines. Black lines represent a background sensor placed 30 cm away from the subject. V/rtHz = voltage over the square root of Hertz; Hz = Hertz

Test 1 and Test 2 data are plotted in Figure 1 along with a background sensor (30 cm away from the subject). Test 2 shows increased peaks compared to Test 1, especially at 3.5 Hz and 9 Hz, consistent with the magnetic field generated by the human subject. There is consistent brain activity from 2.5 Hz to 12.5 Hz. The right-sided brain sensor showed an increased signal compared to the left. The subject is right-handed and demonstrates a larger change from baseline in the left brain during the recording process. There is a clear change from background signal in all three sensors compared to no human subject (Test 2 versus Test 1).

**Figure 2:**
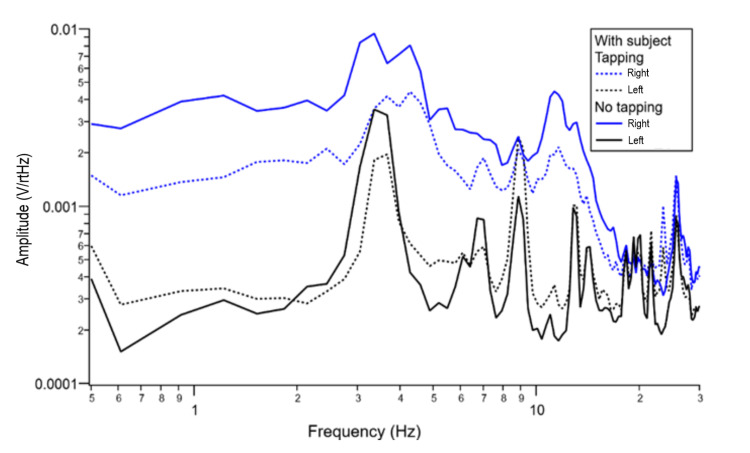
Human subject testing with subject performing movement (tapping) in Test 3 versus no purposeful movement in Test 4. Blue lines represent the right brain sensors. Black lines represent the left brain sensors. Tapping experimentation (Test 5) indicated by dotted lines. V/rtHz = voltage over the square root of Hertz; Hz = Hertz

In Test 3, the human subject was then instructed to tap on the right leg with the right index finger in a roughly consistent time interval of approximately 2 seconds. Sensor data from this testing is represented in Figure 2.

Right finger tapping resulted in negative V/rtHz activity sensed from the right hemisphere and positive activity from the left hemisphere (Figure 2). It would be expected that the sensed brain activity be opposite of the side of body movement; however, this inconsistency was due to the reverse polarity of the sensor used, as discussed. This change occurred at multiple frequencies but especially from 4.5 Hz to 11 Hz. The data also demonstrates a left hemisphere change in relation to right-sided tapping.

**Figure 3:**
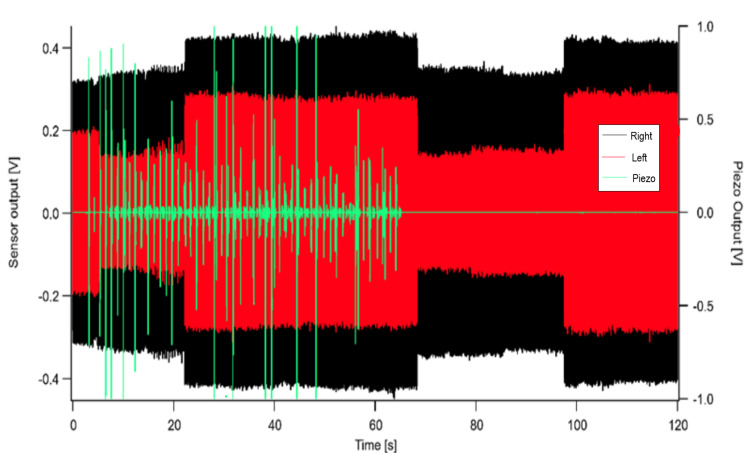
Test 5 time-domain data sensor recording of tapping and piezo stimulation recording. Piezo device stimulation is indicated by green spikes. V = voltage; s = seconds

Figure 3 shows time-domain data for the duration of the 120 seconds Test 5 (tapping on a device located on the subject’s right leg a total of 50 times followed by rest). As seen in the results, the piezo device stimulation by the subject’s right index finger is represented by green spikes for the first 60 seconds followed by 60 seconds of no movement.

**Figure 4:**
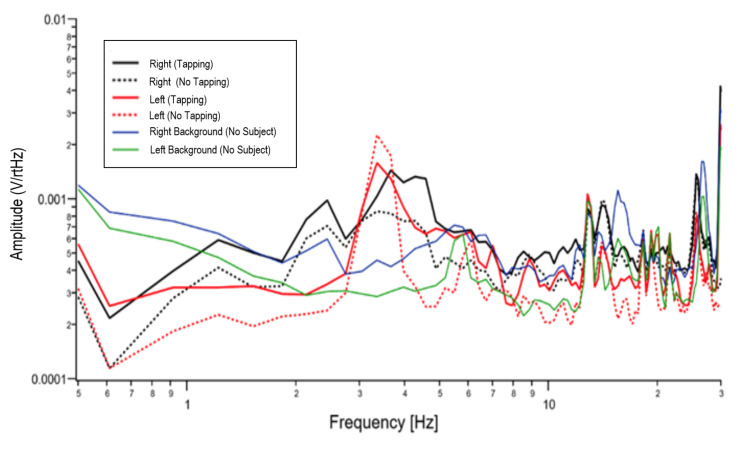
Test 5 comparison of tapping versus no tapping on piezo electric switch. Black lines represent right sided recording. Red lines represent left sided recording. Solid blue line indicates right background recording. Solid green line indicates left background recording. V/rtHz = voltage over the square root of Hertz; Hz = Hertz

The frequency response for Test 5 is found in Figure 4. Similar results to that of Test 3 are seen. Right finger tapping showed a cumulative increased V/rtHz from 3.5 Hz to 11 Hz on the right side and increased V/rtHz on the left side from 4 Hz to 11 Hz (similar to Test 3). There is a change in the shape of the signal from baseline on both sides of the brain recording.

There are changes from baseline without a subject and changes from tapping to no tapping. There are different changes in different frequency domains between the right brain sensor and the left-brain sensor. The recording of the brain activity sampling rate is 5 kS/s or 200 µsec resolution. The sensor has a bandwidth of 30 kHz, and with the 2 kHz low pass anti-aliasing filter with the response time of 500 µsec, demonstrating the ultrafast recording of the magnetic field.

**Figure 5:**
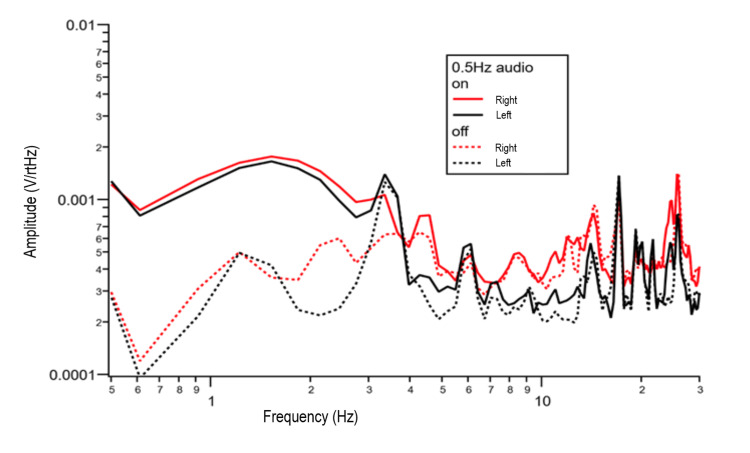
Test 7 data recording subject while piezo crystal emits sound. Sound on (solid lines) versus sound off (dotted lines). Red lines indicating right sided measurement. Black lines representing left sided measurement. V/rtHz = voltage over the square root of Hertz; Hz = Hertz

Auditory stimuli testing in the subject was measured in Test 7. As described in the Methods, a non-magnetic source was used to emit sound with the subject in the room with sensors in place. Figure 5 shows that with 0.5 Hz audio, there is a change in shape and increased voltage activity in the region of 0 to 3 Hz on the left side of the brain and 0 to 4 Hz on the right side of the brain. After this initial increase, the activity equalizes in signal shape and voltage at higher Hz.

**Figure 6:**
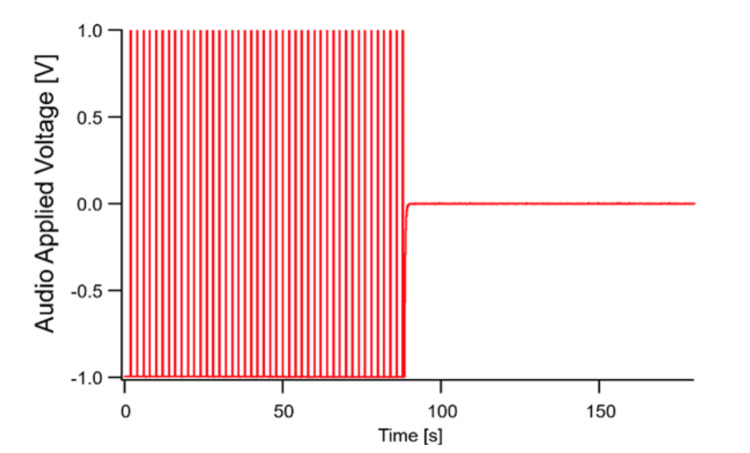
Time domain data of audio applied voltage for Test 7. V = voltage; s = seconds

Figure 6 shows the audio applied voltage for Test 7 as a representation over time. Audio applied voltage was present for approximately 90 seconds, followed by a period of no voltage.

## Discussion

The normal physiological activity of the human brain involves the movement of ions and proteins, which carry an electrical charge [7]. All electrical charge generates an electromagnetic field (EMF) which can be measured with the appropriately tuned tools [3]. As demonstrated in the past by Carson et al., it is possible in a non-contact fashion to passively measure these signals from living hippocampal tissue in the mice models [4]. The next step in this discovery was to assess if it is possible to continuously measure these signals through the human scalp and skull in the standard environment. Our results demonstrate that this is possible. The generation of EMF signals in the brain while generating movement and while hearing an auditory stimulus show that these signals can be captured from a distance in real-time. All of this was accomplished in a continuous, non-invasive, non-contact manner through the entire brain as the brain generated changes in its intrinsic electromagnetic field.

Primary current flows along axons of the cortex, driven primarily by excitatory and inhibitory postsynaptic potentials (excitatory postsynaptic potential [EPSPs] and inhibitory postsynaptic potential [IPSPs], respectively) [7]. The summation of large amounts of action potentials and their parallel orientation is thought to be the basis of the magnetic field [8]. Summation across the space of the magnetic field is more likely when cells have a similar orientation [9]. Therefore, we recorded brain activity from areas known to have large areas of cells working in conjunction. We selected the pre-motor region (frontal lobe), motor (posterior frontal lobe, motor cortex), and post-motor regions (parietal lobe) of the brain. Moreover, since the specific neurons, Betz cells, located in the primary motor cortex, are one of the largest in the central nervous system, these were the target for initial testing. The EMF measured corresponds to brain activity, as demonstrated in our results. The act of movement and the reception of sound resulted in observable changes in the EMF of the human subject.

**Figure 7:**
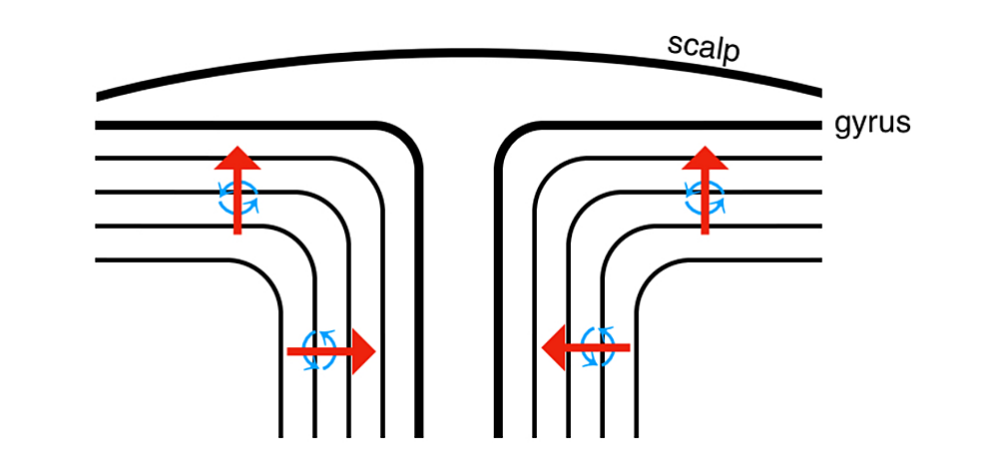
Illustrative representation of a cross section of brain demonstrating neuronal tissue and magnetic fields generated. Red arrows depict the direction of electrical current generated by the neurons. Blue depicts the magnetic field generated by the neuronal tissue.

Figure 7 shows a representation demonstrating the neuronal tissue and magnetic fields generated. EEG would detect the electrical current from the gyral surface but not the sulcus angle. Conversely, the magnetic field of the gyral surface would be invisible to magnetoencephalography (MEG) sensors placed perpendicular to the scalp but not others. Signals from the gyral angle would not be visible to sensors placed perpendicular to the scalp. However, sensors along other parts of the scalp do pick up the signal from those additional magnetic fields generated at the angle of the gyrus.

Having proprietary sensors with enough sensitivity to measure these signals was essential to this study. Moreover, appropriate shielding to reduce any potential interference was also paramount. Metallic shielding created with Mu-metal sheets was used in these trials to block any potential outside EMF interference. Mu-metal is a nickel-iron ferromagnetic alloy that is commonly used for shielding electronic equipment and is effective against low-frequency magnetic fields. Mu-metal achieves this property due to its high magnetic permeability resulting in its ability to absorb magnetic energy [5]. In this preliminary testing, we utilized Mu-metal to surround the subject’s head to minimize interference from their surroundings, the earth’s magnetic field, and from their own extra-cortical magnetic sources. We also placed a background sensor 30 cm away from their head.

Similarly, even sound sensed by the brain can be recorded, as seen in Figure 5. Discernable changes in waves were seen when sound was emitted by the piezoelectric device. In future studies, it may be possible to further delineate the transmitted information to localize and functionally map magnetic fields generated by individuals.

Limitations of our study are the small sample size of subjects and limited types of testing. Further experimentation is being undertaken to include more human subjects and to expand the variety of tests and will be published separately. Reproducibility is limited due to the proprietary nature of the sensors being used. Future studies could compare and contrast the various modalities discussed with our method, including EEG and MEG.

## Conclusions

We have shown that it is possible to measure dynamic living human brain electromagnetic fields in a non-contact, non-invasive, and continuous manner through the skull and scalp in a standard environment. This was demonstrated by having subjects perform a motor movement (tapping) while measuring and recording the resultant EMF activity using proprietary ultra-low noise and high sensitivity sensors placed around the head. Separately, an auditory stimulation resulted in EMF activity that was also measured and recorded. These dynamic signals were differentiated from the background human brain and extraneous activity.

## References

[1] Darvas F, Pantazis D, Kucukaltun-Yildirim E, Leahy RM (2004). Mapping human brain function with MEG and EEG: methods and validation. NeuroImage.

[2] Buxton RB (2013). The physics of functional magnetic resonance imaging (fMRI). Rep Prog Phys.

[3] Hales CG (2017). The origins of the brain’s endogenous electromagnetic field and its relationship to provision of consciousness. Biophysics of Consciousness: A Foundational Approach. World Scientific.

[4] Carson TA, Ghanchi H, Toor H, Majeed G, Wiginton JG 4th, Zhang Y, Miulli DE (2018). Novel method of non-contact remote measurement of neuronal electrical activity. Cureus.

[5] (2022). MagneticShiled Co.: MuMETAL® technical data. https://www.magnetic-shield.com/mumetal-technical-data/.

[6] Zhang Z, Moore J (2011). New significance test methods for fourier analysis of geophysical time series. Non Proc Geop.

[7] Olejniczak P (2006). Neurophysiologic basis of EEG. J Clin Neurophy.

[8] Hari R, Parkkonen L, Hämäläinen M (2015). Basic principles of magnetoencephalography. Elsevier.

[9] Singh SP (2014). Magnetoencephalography: basic principles. Ann Indian Acad Neurol.

